# Risk of Inflammatory Bowel Disease in Patients with Chronic Myeloproliferative Neoplasms: A Danish Nationwide Cohort Study

**DOI:** 10.3390/cancers12092700

**Published:** 2020-09-21

**Authors:** Marie Bak, Tine Jess, Esben Meulengracht Flachs, Ann-Dorthe Zwisler, Knud Juel, Henrik Frederiksen

**Affiliations:** 1 Department of Haematology, Zealand University Hospital, University of Copenhagen, 4000 Roskilde, Denmark; 2 Department of Epidemiology Research, Statens Serum Institut, 2300 Copenhagen, Denmark; tjs@ssi.dk; 3 Department of Occupational and Environmental Medicine, Bispebjerg Hospital, University of Copenhagen, 2400 Copenhagen, Denmark; esben.meulengracht.flachs@regionh.dk; 4 Danish Knowledge Centre for Rehabilitation and Palliative Care, University of Southern Denmark and Odense University Hospital, 5800 Nyborg, Denmark; ann.dorthe.olsen.zwisler@rsyd.dk; 5 National Institute of Public Health, University of Southern Denmark, 1455 Copenhagen, Denmark; knj@sdu.dk; 6 Department of Haematology, Odense University Hospital, 5000 Odense, Denmark; henrik.frederiksen@rsyd.dk; 7 Department of Clinical Epidemiology, Aarhus University Hospital, 8200 Aarhus, Denmark

**Keywords:** inflammatory bowel disease, myeloproliferative disorders, comorbidity, registry-based, cohort study

## Abstract

**Simple Summary:**

We wanted to investigate the risk of inflammatory bowel disease (IBD) in patients with Philadelphia-negative chronic myeloproliferative neoplasms (MPNs), since up to 50% of these patients experience gastrointestinal symptoms and several studies have suggested an association between hematological cancers and IBD. We included ∼8000 patients and ∼80,000 sex- and age-matched, non-MPN comparisons from the general population, and found that MPN patients were two to three times more likely to develop IBD, but the absolute risk of IBD was modest. In addition, MPN patients were also 40% more likely to have a prior diagnosis of IBD. Our results pose intriguing questions about the causal pathways linking MPN and IBD, which may include genetic, treatment-related and immune-mediated factors. Moreover, it shows that abdominal symptoms in MPN patients may not only be caused by an enlarged spleen or treatment side-effects. Conversely, persistent leucocytosis and/or increased platelets in IBD patients may reflect concomitant MPN.

**Abstract:**

An association between hematological cancers and inflammatory bowel disease (IBD) has previously been suggested, but the risk of IBD in patients with myeloproliferative neoplasms (MPNs) is unknown. We conducted a nationwide population-based cohort study using Danish registries, to estimate the risk of IBD in individuals diagnosed with essential thrombocythemia, polycythemia vera, myelofibrosis or unclassifiable MPN during 1994–2013. MPN patients were matched 1:10 with sex- and age-matched comparisons. Everyone was followed until a diagnosis of IBD, death/emigration, or 31 December 2013. The risk of IBD overall and according to MPN subtype was calculated using Cox regression and presented as hazard ratios (HRs) with 95% confidence intervals (CI). Of 8207 MPN patients followed for 45,232 person-years, 80 were diagnosed with IBD (61 ulcerative colitis, 19 Crohn’s disease). The rate of IBD per 1000 person-years was 1.8 (95% CI:1.4–2.2) in patients vs. 0.8 (95% CI:0.7–0.8) in comparisons, and the absolute 10-year risk of IBD was 0.8% (95% CI:0.6–1.0) in patients vs. 0.4% (95% CI:0.4–0.5) in comparisons. The HR of IBD was 2.4 (95% CI:2.1–2.9) with similar HRs for ulcerative colitis and Crohn’s disease. MPN subtype risks varied from 2.1 (95% CI:1.6–2.7) to 2.8 (95% CI:2.1–3.7). Our unselected cohort study showed a more than 2-fold increased risk of IBD in MPN patients.

## 1. Introduction

For inflammatory bowel disease (IBD), comprising ulcerative colitis (UC) and Crohn’s disease (CD), the etiological causes are not entirely known, but in particular, chronic inflammation is a key feature [1,2]. The diseases are characterized by continuous perturbation of the immune system, which leads to inflammation of the gastrointestinal tract. Studies show that patients with IBD are at increased risk of various chronic conditions, such as cardio-vascular diseases, osteoporotic fractures, and other immune-mediated diseases [3,4,5,6,7]. In addition, the risk of gastrointestinal cancers is increased, as is the risk of some extra-intestinal malignancies, including hematological cancers [8,9,10,11,12,13,14,15]. Conversely, the subsequent risk of IBD in patients with hematological cancers has not been studied.

Chronic myeloproliferative neoplasms (MPNs) are chronic hematological cancers that arise due to clonal hematopoietic stem cell proliferation in the bone marrow. The classic Philadelphia chromosome negative MPNs include essential thrombocythemia (ET), polycythemia vera (PV), and myelofibrosis (MF) [16]. Increased platelets are primarily seen in ET, and increased red blood cell levels with or without leuko- and/or thrombocytosis are observed in PV. In MF, which is the more advanced form of MPN, characterized by bone marrow fibrosis and dismal prognosis, patients present with elevated leucocytes and/or platelets, or pancytopenia. Overall, patients with MPN have decreased survival compared to the general population [17]. The etiology of MPNs remains somewhat unknown, but acquired somatic MPN driver mutations in *JAK2*, *MPL* or *CALR* that alter the JAK-STAT signaling pathway are found in most patients [16].

Although autoimmune diseases and conditions mediated by inflammation have been reported in patients with MPN [18,19,20,21] and cross sectional surveys have documented that abdominal discomfort and pain are present in approximately 45–50% of the patients [22], the risk of IBD in these patients is unknown.

Chronic inflammation seems to be involved in the pathophysiology of both IBD [1,2] and MPNs [23] and it contributes significantly to the symptom burden as well [1,24,25]. Moreover, shared genetic predisposition has been demonstrated in MPN [26] and inflammatory diseases, including IBD [27,28,29,30]. Given this background, the aim of the present study was to investigate the risk of IBD in patients with MPN compared to sex- and age-matched individuals from the general population.

## 2. Results

**Figure 1:**
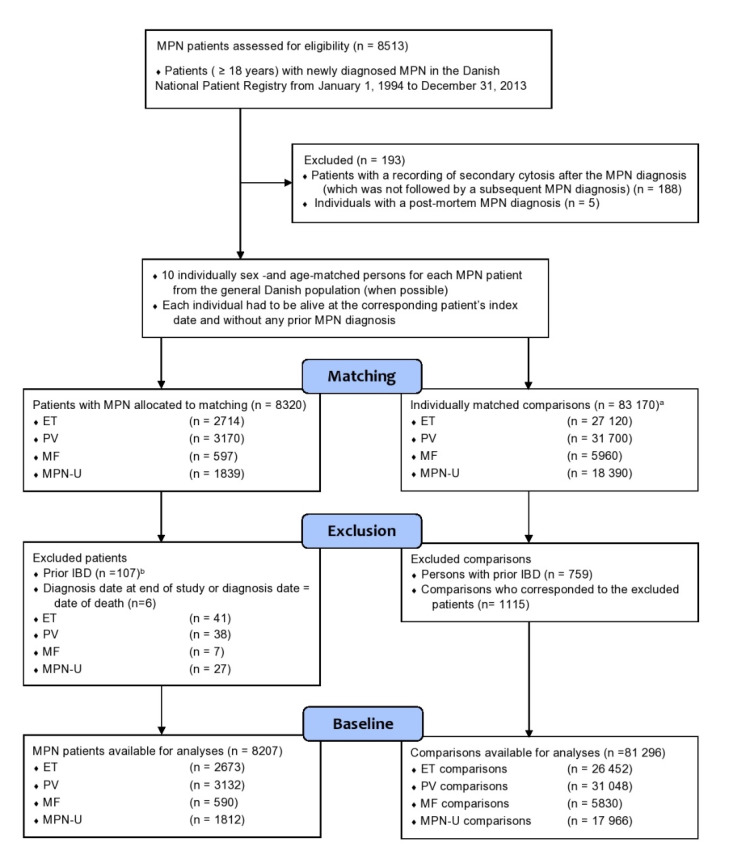
Flowchart of included MPN (myeloproliferative neoplasm) patients (ET (essential thrombocythemia), PV (polycythemia vera), MF (myelofibrosis), and MPN-U (unclassifiable MPN)) and sex- and age-matched comparisons. ^a^ For a few patients <10 comparisons could be matched due to old age. ^b^ The prevalence of IBD (inflammatory bowel disease) at the time of MPN diagnosis is shown in Table 1.

In total, 8513 patients were eligible for inclusion in the study (Figure 1). Of those, 193 were excluded (188 persons with secondary blood cytosis as the last recorded code in the Danish National patient Registry (DNPR) and five persons who had a postmortem diagnosis). Of the 8320 MPN patients who were allocated to matching, 107 patients (ET = 40, PV = 37, MF = 5, and unclassifiable MPN (MPN-U) = 25) were excluded due to prior IBD, as were 759 of the comparisons. We subsequently also excluded six patients (and their matched comparisons) due to either date of diagnosis at the end of 2013 or a diagnosis date equal to date of death. This resulted in 8207 MPN patients and 81,296 comparisons available for analysis.

During a total risk time of 45,232 person-years, with a mean follow-up time of 5.5 years (SD = 4.7), 80 MPN patients were diagnosed with IBD (37 patients with ET, 28 patients with PV, 1 patient with MF, and 14 patients with MPN-U). Of those, 9 patients (11%) were diagnosed with both UC and CD; 55 patients with UC (69%), and 16 patients with CD (20%). Among the comparisons, 380 were diagnosed with IBD during the study period. A total of 50 comparisons (13%) were diagnosed with both UC and CD, 241 (63%) with UC, and 89 (23%) with CD, respectively. The characteristics of the patient and comparison cohorts, including number of IBD events, are shown in Table 1.

The overall rates of IBD per 1000 person-years were 1.8 (95% CI 1.4 to 2.2) for patients with MPN and 0.8 (95% CI 0.7 to 0.8) for comparisons. The absolute 10-year risks were 0.8% (95% CI 0.6 to 1.0) and 0.4% (95% CI 0.4 to 0.5), respectively. The 1-year, 3-year, 6-year, and 10-year risks of IBD are shown in Table 2.

The risk of IBD was significantly increased among patients with MPN, with HRs between MPN patients and matched comparisons of 2.4 (95% CI 2.1 to 2.9) for IBD overall, 2.6 (95% CI 2.1 to 3.2) for UC, and 2.4 (95% CI 1.7 to 3.4) for CD. Stratified by MPN subtype, the highest risk of IBD was seen among patients with ET (HR 2.8; 95% CI 2.1 to 3.7). Comparable HRs were observed among patients with PV (HR 2.1; 95% CI 1.6 to 2.7) and MPN-U (HR 2.2; 95% CI 1.3 to 3.7). In patients with MF, only one person was diagnosed with IBD. The risk of UC and CD displayed similar patterns as for IBD overall across the different MPN subtypes, with the highest risk observed for patients with ET. The HRs of UC and CD, following a diagnosis of ET, PV, or MPN-U, are shown in Table 3.

The risk of IBD was significantly increased within the first year and more than five years after the MPN diagnosis ([HR 0–1 year 4.6; 95% CI 2.8 to 7.6]; [HR > 5 years 3.0; 95% CI 2.1 to 4.2]), whereas the risk was only slightly higher than in comparisons one to five years following the MPN diagnosis. HRs adjusted for time since index date are shown in Table 3, but we refrained from calculating the adjusted risk of UC and CD by MPN subtypes due to low numbers. In the sensitivity analyses confined to individuals with more than one recording of IBD, the HRs of IBD were also increased, with HRs of 2.1 (95% CI 1.7 to 2.6) for all MPNs, 2.8 (95% CI 2.0 to 3.9) for ET, 1.7 (95% CI 1.2 to 2.3) for PV and 1.8 (95% CI 0.9 to 3.7) for MPN-U.

In the opposed case-control study, assessing the odds of prior IBD among patients with MPN and their comparisons, we found that patients with MPN were also significantly more likely to have a personal history of IBD than the matched comparisons at the time of diagnosis, with an odds ratio of 1.4 (95% CI, 1.1 to 1.7) (Table 4).

## 3. Discussion

In this nationwide cohort study of more than 8000 patients with MPN followed for 45,232 person-years, we found a 2.4-fold (95% CI 2.1 to 2.9) increased risk of IBD in MPN patients when compared to matched non-MPN comparisons. To our knowledge, this is the first study to investigate the risk of IBD during the course of ET, PV, MF, and MPN-U. Additionally, we also found that more patients than comparisons had a personal history of IBD (OR 1.4; 95% CI 1.1 to 1.7). The association was comprehensively investigated using large nationwide cohorts of unselected patients with MPN and individually sex- and age-matched comparisons from the general population. We used data from virtually complete population-based national registries, in which the recorded diagnosis codes for both IBD and MPNs have been validated [31,32].

We found an increased risk of IBD among patients with ET, PV, and MPN-U, but the risk was different over time across the different MPN subtypes and the absolute 10-year risks of IBD were low both for patients with MPN and comparisons—in particular among patients with MF, as only one event was recorded. Up to half of patients with MPN can experience abdominal discomfort, which is most often caused by an enlarged spleen and/or treatment side effects [22]. Therefore, physicians may not have attributed abdominal symptoms to IBD in some MF patients, as the disease and symptom burden is often very severe in these patients. Conversely, most IBD diagnoses were recorded among patients with ET. Since blood cytosis caused by other conditions may mimic MPNs, and the distinction between the MPN subtypes can also be difficult at times, we cannot exclude the misclassification of a few patients due to diagnostic ambiguity [33].

The study has some additional limitations. Firstly, detection bias leading to the overrepresentation of co-incident IBD events during the diagnostic phase and clinical follow-up of the MPNs may influence the results, since the observed risk of IBD was highest within the first year following MPN diagnosis. An alternative (hypothetical) explanation for the short latency between the onset of MPNs and IBD could also be extensive (untreated) inflammation at the time of MPN diagnosis, leading to the evolvement of the diseases in close proximity.

Secondly, we cannot exclude that some diagnosis codes have been incorrectly reported, and defining IBD as records of a single diagnosis could have led to misclassification in a few cases. Nevertheless, when using a pathology database as reference, the completeness of IBD recording in the DNPR has previously been shown to be high (94%), with estimated validity (fulfilling the diagnostic criteria) of 97% for CD and 90% for UC [32]. Further, when requiring two records for case definition, we still found an increased risk of IBD in patients with MPN. This approach has been used by others, since using two records of IBD diagnosis provide more valid incidence estimates [34]. It is also well described that IBD sub-classification is altered in up to 10% of the patients during the course of the disease. Since we defined the first recorded IBD diagnosis code as outcome, our results may reflect the overall IBD risk more than the exact rates of UC and CD. However, the coding precision of the IBD diagnoses in the DNPR is unlikely to be different for MPN patients and comparisons.

Thirdly, due to the registry-based study design, detailed clinical information such as smoking status, gene mutations, or MPN treatments was not available. This precluded us from investigating whether these factors influenced the risk of IBD.

Although the risk of IBD has not previously been compared in patients with MPN and matched comparisons, the association between autoimmune diseases and MPNs has been proposed by others [35,36,37]. Several studies have investigated autoimmune and chronic inflammatory diseases occurring prior to an MPN diagnosis. Some studies have reported that the risk of MPN is increased for persons who have a personal history of autoimmune diseases [35,36,37], but few studies have reported results on antecedent IBD in patients with MPN [35,37]. When investigating 1017 patients with ET, PV, MF, mastocytosis, hypereosinophilic syndrome, or histiocytosis from the U.S., Anderson and colleagues found that antecedent CD was associated with a two-fold increased risk of MPN, however, they did not find a significant association to prior UC [35]. Similarly, in a nationwide Swedish study of 11,039 ET, PV, MF, and MPN-U patients and 43,550 sex- and age-matched comparisons, Kristinsson and colleagues found an odds ratio of 1.8 (CI 95% 1.3 to 3.0) for MPN following a personal history of CD and 1.3 (CI 95% 0.8 to 2.1) for UC [37].

Conversely, as mentioned previously, increased risks of various hematological cancers have also been reported for patients with IBD, but different risks have been observed among patients with CD and UC [8,9,10,11,12,13,14]. An ECCO guideline/consensus paper from 2015 pointed to lymphoma as more likely occurring in patients with CD, and leukemia more likely occurring in patients with UC [8]. However, the results are inconclusive, and recently published data again show diverging results of the risk for hematological malignancies in patients with IBD [13,14]. Of note, the specific relationship with MPNs has been sparsely investigated in patients with IBD, since MPNs have not been included as myeloid malignancies in all previous studies [12,14,15]. Nevertheless, Cheddani and colleagues found that the risk of myeloid malignancies (chronic myeloid leukemia, acute myeloid leukemia, ET, PV, and MF), was increased 2-fold among 844 elderly patients with IBD from France compared to that observed in a population-based cancer registry [14]. This increased risk of hematological cancers among patients with IBD has previously been correlated to the use of immune-modulating treatments, but the results are ambiguous [8,14,38,39,40].

Since we now find both an increased risk of IBD in patients with an already established diagnosis of MPN and an increased OR of IBD prior to the MPN diagnosis, converging causes of MPN and IBD must be considered. The development of these apparently disparate diseases in the same patient likely involve complex interactions between multiple environmental, treatment-related, and inflammation- and immune-mediated factors. Common genetic susceptibility may also contribute, and genome-wide association studies indicate overlap in genes associated with both diseases (e.g., the *JAK2* and *STAT3* genes) [26,27,28,29,30]. The haplotype 46/1 (“GGCC”), a specific constitutional *JAK2* gene haplotype that increases the risk of MPN (including both increased susceptibility to develop *JAK2* mutated MPNs and less often non-*JAK2* mutated MPNs) [26], is also found to be a predisposition factor for IBD [28,30]. For instance, as the specific *JAK2* rs10758669 single nucleotide polymorphism, part of the haplotype 46/1, is shown to be associated with both CD and UC [27,28,30]. Strengthening the notion of shared genetic predisposition, Kuriakose and colleagues showed that the *JAK2* V617F mutation was present in four of 23 patients with IBD who also had increased erythrocyte or platelet counts [15]. Further, in patients with MPN, a correlation between *JAK2* V617F positivity and prior autoimmune disease has been observed [36]. Intriguingly, treatment with JAK inhibitors for patients with IBD is currently under investigation in phase II and III clinical trials [41].

As mentioned, chronic systemic inflammation is also a common denominator, with similar alterations displayed for different immune modulatory mechanisms leading to inappropriate immune regulatory responses and crosstalk between inflammation, hypoxia, and angiogenesis [1,23,42,43,44]. In both IBD and MPNs, increased levels of circulating inflammatory cytokines, like tumor necrosis factor-α, IL-6, and IL-1β, and the accumulation of reactive oxygen and nitrogen species due to oxidative stress have been observed [1,23,45,46]. In addition, stress responses involving the Janus kinase/signal transducer and activator of transcription (JAK/STAT) and the anti-inflammatory nuclear factor-erythroid 2-related factor-2 (Nrf2) pathways appear to be altered. Under normal circumstances, the Nrf2 regulates the expression of cytokines, which protects cells against oxidative stress, but attenuated Nrf2 signaling due to downregulation of the coding gene has been observed in MPNs [47]. The depletion of Nrf2 has also been associated with the expansion of hematopoietic stem and progenitor cells [48] and Nrf2 signaling also appears to be involved in IBD [49,50,51]. However, MPN and IBD are likely not only interlinked by chronic systemic inflammation, as the risk of IBD among patients with MF, per se, would be highest, since this disorder is characterized by severe inflammation [23].

The JAK/STAT pathway is also altered in both MPNs and IBD. It facilitates the intracellular signaling cascade of cytokines, thus leading to changes in the expression of genes that are important for cell proliferation and survival. The pathway is pivotal in MPNs, as constitutively activation of the JAK/STAT signaling (by e.g., the *JAK2* V617F mutation) leads to the abnormal growth of myeloid cells [52]. Studies also show that changes in the JAK/STAT signaling are involved in autoimmunity [53]. Finally, overlap in the presence of concurrent diseases driven by chronic inflammation and oxidative stress such as cardiovascular diseases, osteoporotic fractures, immune-mediated eye diseases and autoimmune diseases has been observed among both patients with IBD [3,4,5,6,7] and patients with MPN [18,19,20,21].

## 4. Materials and Methods

### 4.1. Data Sources

We obtained data from two national population-based registries, covering the entire nation of approximately 5.7 million persons: The Danish Civil Registration System (CRS) and the DNPR [54,55]. The CRS is an administrative population-based registry that records (continuously updated) information on everyone residing in Denmark, including vital status and date of death or emigration [54]. Unique personal civil registration numbers, assigned at birth or immigration, enable individual-level linkage of data between the different Danish registries. Since all persons in Denmark have equal access to income-independent free healthcare, and data on healthcare services provided at hospitals nationwide are by law recorded in the DNPR, we could construct a nationwide cohort of patients with MPN. The DNPR contains information on dates of hospital admissions and out-patient visits as well as all diagnostic codes according to the World Health Organization’s International Classification of Diseases (8th revision [ICD-8] from 1977–1993 and 10th revision [ICD-10] thereafter) [55]. Patients’ civil registration numbers also allowed us to track a cohort of sex- and age-matched comparisons from the general population through the CRS. Information on IBD diagnoses for both patients and comparisons was also obtained from the DNPR.

### 4.2. Cohorts of Patients and Comparisons

We included all persons ≥18 years who were newly diagnosed with MPN in an in-patient admission or out-patient hospital specialist clinic between 1 January 1994 and 31 December 2013. Patients with a recording of the following MPN subtypes according to the ICD-10 codes were included: ET (D47.3, D75.2), PV (D45), MF (D47.4, C94.5), and MPN-U (D47.1). To avoid diagnosis ambiguity, patients who had a recording of an unspecified myelodysplastic syndrome/chronic myeloproliferative disease were not eligible for inclusion. Subsequently, individuals for whom a postmortem MPN diagnosis was recorded, as well as all patients recorded with an MPN diagnosis, who, in addition, had a secondary blood cytosis code (D75.1) as the last recorded code in the DNPR, were excluded. This was done since few individuals with reactive blood cytosis could have been misinterpreted and thus misclassified as MPNs when the initial diagnostic blood work was performed. For each patient, we included 10 randomly selected comparisons without MPN from the general population. The comparisons were matched on sex and age (birth month and year) and had to be alive at the MPN diagnosis date of the corresponding patient. Every comparison was assigned a study entry identical to the date of MPN diagnosis for the patient for whom they were selected. This date was defined as the index date for both patients and comparisons. Prior to analysis, we excluded persons who did not contribute with risk time (persons who had an MPN diagnosis date equal to the date of death or patients who had a date of diagnosis at the ultimate end of 2013). Everyone with a prior recording of IBD in the DNPR was also excluded. No other exclusion criteria besides the above mentioned were applied for patients or comparisons.

### 4.3. Cohorts Follow-up, Statistical Analyses, and Ethics

Patients and comparisons were followed from index date and until the earliest of the following occurred: a first-time IBD diagnosis, emigration, death, or end of study (31 December 2013). IBD events were defined as an in-patient or out-patient hospital clinic visit with a diagnosis code of either UC (ICD-10: K51) or CD (ICD-10: K50). Individuals who had a recording of both UC and CD codes during the study period were included in the overall IBD risk analysis from the date of the first of these two diagnoses. Conversely, these patients were excluded from the separate UC and CD analyses.

The risk of IBD in patients with MPN versus matched comparisons was estimated using Cox regression models, and the results were presented as hazard ratios (HRs) with 95% confidence intervals (CIs). We calculated HRs of IBD for all MPN patients and stratified by MPN subtype (ET, PV, MF, and MPN-U). Subsequently, the HRs of UC and CD was calculated separately. If five or fewer IBD events occurred, the risk of IBD was not calculated. To test the robustness of the results, we performed sensitivity analyses where a confirmed IBD diagnosis required recording of at least two subsequent IBD diagnosis codes.

The proportional hazard assumption was tested graphically for IBD and was found to be violated. We therefore also included an analysis adjusting for time since the index date. The hazard assumption in this model was not violated. No other adjustments were made.

Finally, in a separate matched case-control analysis, we calculated the odds of being diagnosed with IBD before the index date, using the same patients and comparisons as in the cohort study, but without excluding persons who had a prior IBD diagnosis.

All analyses were carried out using the SAS version 9.3 (SAS Institute Inc., Cary, NC, USA) and R version 3.4.4 software systems. The study was approved by the Danish Data Protection Agency (REG-82-2013). Approval from the ethics committee is not mandated for registry-based studies without direct patient contact according to Danish law.

## 5. Conclusions

In conclusion, our study of more than 8000 patients with MPN shows a 2.4-fold increased risk of IBD and a modest absolute risk of IBD in patients with MPN compared to the general population. IBD was also more commonly seen in MPN patients prior to their hematological malignancy. This points towards shared pathophysiological mechanisms that merit further investigation.

## Figures and Tables

**Table 1:** Demographic characteristics of nationwide cohorts of patients 1994–2013 with myeloproliferative neoplasms (essential thrombocythemia, polycythemia vera, myelofibrosis, and unclassifiable myeloproliferative neoplasms) and sex- and age-matched comparisons without myeloproliferative neoplasms.

	MPNs, Total		ET		PV		MF		MPN-U	
Number of Patients	Patients	Comparisons	Patients	Comparisons	Patients	Comparisons	Patients	Comparisons	Patients	Comparisons
Men	3780	37,454	950	9401	1601	15,872	328	3241	901	8940
Women	4427	43,842	1723	17,051	1531	15,176	262	2589	911	9026
Total	8207	81,296	2673	26 452	3132	31,048	590	5830	1812	17,966
**Mean age at MPN diagnosis (SD)**	67.0 (14.3)	67.0 (14.3)	64.4 (15.4)	64.3 (15.3)	66.4 (13.9)	66.4 (13.9)	71.1 (11.9)	71.1 (11.9)	70.6 (13.2)	70.60 (13.2)
**Year of MPN diagnosis/index date**										
1994–1998	1831	18,202	444	4412	866	8613	175	1741	346	3436
1999–2003	1797	17,831	571	5661	676	6703	91	902	459	4565
2004–2008	2185	21,632	823	8144	810	8024	77	755	475	4709
2009–2013	2394	23,631	835	8235	780	7708	247	2432	532	5256
**Risk time, years**										
Total, years	45 232	504,818	16,487	166,457	20,249	205,861	1477	29,156	7019	103,344
Mean follow-up Time, years (SD)	5.5 (4.7)	6.2 (4.9)	6.2 (4.7)	6.3 (4.8)	6.5 (4.9)	6.6 (5.1)	2.5 (3.3)	5.0 (5.0)	3.9 (3.8)	5.8 (4.5)
**Number of IBD events ^a^**										
Total, n	80	380	37	126	28	163	1	25	14	66
UC, n (%)	55 (68.8%)	241 (63.4%)	26 (70.3%)	78 (61.9%)	20 (71.4%)	104 (63.8%)	1 (100.0%)	15 (60.0%)	8 (57.1%)	44 (66.7%)
CD, n (%)	16 (20%)	89 (23.4%)	7 (18.9%)	32 (25.4%)	6 (21.4%)	37 (22.7%)	0 (0.0%)	6 (24.0%)	3 (21.4%)	14 (21.2%)
**IBD rate pr. 1000 PYR (95% CI)**	1.8 (1.4–2.2)	0.8 (0.7–0.8)	2.2 (1.6–3.0)	0.8 (0.6–0.9)	1.4 (0.9–2.0)	0.8 (0.7–0.9)	0.7 (0.0–3.2)	0.9 (0.6–1.2)	2.0 (1.2–3.5)	0.6 (0.5–0.8)

^a^ Note: Includes patients and comparisons with recorded diagnosis codes for UC (ulcerative colitis), CD (Crohn’s disease), or both codes in the DNPR (Danish National patient Registry). MPN—myeloproliferative neoplasms; ET—essential thrombocythemia; PV—polycythemia vera; MF—myelofibrosis; IBD—inflammatory bowel disease.

**Table 2:** Absolute risks of IBD among patients with myeloproliferative neoplasms (essential thrombocythemia, polycythemia vera, myelofibrosis, and unclassifiable myeloproliferative neoplasms) and their matched comparison cohorts between 1994 and 2013.

Absolute Risks of IBD, % (95% CI)		MPN Patients		Comparisons
MPNs, total *	*n*		*n*	
1-year risk	22	0.3 (0.2, 0.4)	49	0.1 (0.0, 0.1)
3-year risk	35	0.4 (0.3, 0.6)	153	0.2 (0.2, 0.2)
6-year risk	51	0.6 (0.5, 0.8)	255	0.3 (0.3, 0.4)
10-year risk	65	0.8 (0.6, 1.0)	330	0.4 (0.4, 0.5)
**ET**				
1-year risk	11	0.4 (0.2, 0.7)	17	0.1 (0.0, 0.1)
3-year risk	19	0.7 (0.4, 1.1)	47	0.2 (0.1, 0.2)
6-year risk	26	1.0 (0.6, 1.4)	85	0.3 (0.3, 0.4)
10-year risk	28	1.0 (0.7, 1.5)	110	0.4 (0.3, 0.5)
**PV**				
1-year risk	4	0.1 (0.0, 0.3)	19	0.1 (0.0, 0.1)
3-year risk	6	0.2 (0.1, 0.4)	67	0.2 (0.2, 0.3)
6-year risk	11	0.4 (0.2, 0.6)	112	0.4 (0.3, 0.4)
10-year risk	22	0.7 (0.4, 1.0)	138	0.4 (0.4, 0.5)
**MPN-U**				
1-year risk	7	0.4 (0.2, 0.7)	11	0.1 (0.0, 0.1)
3-year risk	9	0.5 (0.2, 0.9)	30	0.2 (0.1, 0.2)
6-year risk	13	0.7 (0.4, 1.2)	45	0.3 (0.2, 0.3)
10-year risk	14	0.8 (0.4, 1.3)	62	0.3 (0.3, 0.4)

* Note: Patients with MF are included in the overall analysis, but the absolute risk is not shown for MF patients, as only one patient was diagnosed with IBD.

**Table 3:** Hazard ratios for IBD among patients with MPN compared to sex- and age-matched comparisons from the general population.

Risk of IBD, Crude HRs (95% CI)	Events, n		MPNs Overall (Including MF)	ET	PV	MPN-U
	**Patients**	**Comparisons**				
IBD	80	380	2.4 (2.1–2.9)	2.8 (2.1–3.7)	2.1 (1.6–2.7)	2.2 (1.3–3.7)
UC	55	241	2.6 (2.1–3.2)	3.0 (2.2–4.3)	2.2 (1.6–3.1)	1.7 (0.9–3.4)
CD	16	89	2.4 (1.7–3.4)	3.2 (1.9–5.3)	2.1 (1.2–3.6)	1.6 (0.4–6.9.1)
			**HRs, Adjusted For Time Since Diagnosis**			
IBD overall						
0–1 years			4.6 (2.8–7.6)	6.4 (3.0–13.8)	2.1 (0.7–6.1)	7.0 (2.7–17.9)
1–3 years			1.4 (0.8–2.4)	2.7 (1.2–5.8)	0.4 (0.1–1.7)	1.4 (0.3–6.2)
3–5 years			1.1 (0.6–2.4)	1.4 (0.5–4.1)	0.6 (0.1–2.5)	2.2 (0.5–9.5)
>5 years			3.0 (2.1–4.2)	2.8 (1.6–5.1)	3.4 (2.0–5.6)	2.7 (0.8–8.9)
UC						
0–1 years			5.3 (3.0–9.3)			
1–3 years			1.7 (0.9–3.2)			
3–5 years			1.3 (0.6–3.1)			
>5 years			2.8 (1.7–4.5)			
CD						
0–1 years			1.9 (0.6–6.6)			
1–3 years			1.5 (0.4–4.9)			
3–5 years			0 (0–Inf)			
>5 years			3.3 (1.6–6.6)			

Note: The UC and CD incidence was calculated in cohorts where individuals with both IBD diagnosis codes had been excluded.

**Table 4:** Number of IBD diagnoses prior to the diagnosis of myeloproliferative neoplasms.

	MPNs			ET		PV		MF		MPN-U	
Number of Persons with an IBD Diagnosis Prior to the Index Date	Patients	Comparisons	Odds Ratio (95% CI)	Patients	Comparisons	Patients	Comparisons	Patients	Comparisons	Patients	Comparisons
Total IBD, (n) ^a^	107	759	1.4 (1.1–1.7)	40	265	37	274	5	60	25	160
UC	76	500	1.3 (0.9–1.8)	28	172	29	183	4	40	15	105
CD	26	200	1.5 (1.2–1.9)	11	74	4	68	1	15	10	43

^a^ Individuals who had a recording of either UC or CD or who had a recording of both UC and CD prior to the MPN diagnosis/index date.
